# Comprehensive therapy program in people with mental disorders after COVID-19

**DOI:** 10.1192/j.eurpsy.2026.11018

**Published:** 2026-07-31

**Authors:** N. O. Maruta, V. Y. Fedchenko, T. V. Panko, I. O. Yavdak, O. E. Semikina

**Affiliations:** Borderline Psychiatry, “Institute of Neurology, Psychiatry and Narcology named after P. V. Voloshyn of NAMS of Ukraine” SI, Kharkiv, Ukraine

## Abstract

**Introduction:**

The mechanisms of the formation of mental disorders after a coronavirus disease are determined by the pathoplastic factors of COVID-19 and the patient’s personal reactions to the disease, and are also associated with the psychogenic impact of stressors of the SARS-COV-2 pandemic.

**Objectives:**

To develop a comprehensive therapy program (pharmaco- and psychotherapy) based on identified targets of therapeutic influence in people with mental disorders who have experienced COVID-19 and have been exposed to the stressors of the SARS-CoV-2 pandemic.

**Methods:**

71 patients with a history of mental disorders who have experienced COVID-19 were examined and made up the main group (F 32.0-32.2, 33.1, 33.2 – 24 patients, F 06.3-06.6 – 23 patients, F 41.1, 41.2, 42.2, 45.3, 48.0 – 24 patients). Clinical-psychopathological, clinical-amnestic, psychometric (the Clinical Global Impression Scale (CGI)) and methods of statistical analysis were applied.

**Results:**

Pharmacotherapy should be rationally directed not only at the specific correction of psychopathological manifestations, but also to include general strengthening, metabolic, neuroprotective, antiparoxysmal treatment. In depressive disorders, preference is given to antidepressants (AD) groups of selective serotonin reuptake inhibitors (SSRIs), selective serotonin and norepinephrine reuptake inhibitors (SSRIsN) and 2nd generation antipsychotics; in mental disorders of organic genesis - AD groups of SSRIs and melatonin receptor agonists; in neurotic, stress-related and somatoform disorders - anxiolytics and AD groups of SSRIs.

The developed psychotherapy consists of 4 stages: stage 1 – informational diagnostic (2-3 days, comprehensive assessment of the clinical and psychopathological state); stage 2 – therapeutic and stabilizing (3-4 individual sessions, use of CBT techniques, including NET); stage 3 – rehabilitation (8-10 sessions within individual and group psychotherapy, use of CBT techniques, art therapy and family psychotherapy); stage 4 – preventive (3-4 sessions within individual and group psychotherapy, use of CBT techniques and family psychotherapy). Each stage of psychotherapy has both the same approaches and a certain structure depending on the mental disorder.

**Conclusions:**

The effectiveness of the overall impact of the developed comprehensive therapy program was confirmed. At the time of discharge from the hospital, the condition according to the CGI-S scale was completely normalized in (9.86 ± 3.54)% of patients, corresponded to a borderline disorder in (26.76 ± 5.25)% of patients, mild – in (50.70 ± 5.93)% of patients, and moderate – in (12.68 ± 3.95)% of patients.

**Disclosure of Interest:**

None Declared

